# Developing a Mobility Protocol for Early Mobilization of Patients in a Surgical/Trauma ICU

**DOI:** 10.1155/2012/964547

**Published:** 2012-12-20

**Authors:** Meg Zomorodi, Darla Topley, Maire McAnaw

**Affiliations:** Chapel Hill School of Nursing, University of North Carolina, Chapel Hill, NC 27599-7460, USA

## Abstract

As technology and medications have improved and increased, survival rates are also increasing in intensive care units (ICUs), so it is now important to focus on improving the patient outcomes and recovery. To do this, ICU patients need to be assessed and started on an early mobility program, if stable. While the early mobilization of the ICU patients is not without risk, the current literature has demonstrated that patients can be safely and feasibly mobilized, even while requiring mechanical ventilation. These patients are at a high risk for muscle deconditioning due to limited mobility from numerous monitoring equipment and multiple medical conditions. Frequently, a critically ill patient only receives movement from nurses; such as, being turned side to side, pulled up in bed, or transferred from bed to a stretcher for a test. The implementation of an early mobility protocol that can be used by critical care nurses is important for positive patient outcomes minimizing the functional decline due to an ICU stay. This paper describes a pilot study to evaluate an early mobilization protocol to test the safety and feasibility for mechanically ventilated patients in a surgical trauma ICU in conjunction with the current unit standards.

## 1. Introduction

As critical care clinicians address the complexities of care in the 21st century, the patient care team must be able to identify areas where patient outcomes can be enhanced. Among the most important interventions to reduce morbidity and mortality is early mobility. Early mobility has been linked to decreased morbidity and mortality [1] as inactivity has a profound adverse effect on the brain, skin, skeletal muscle, pulmonary, and cardiovascular systems [2–4]. Delirium, decubitus ulcers, muscular atrophy, and deconditioning may occur in the immobile patient, as a result of atelectasis, pneumonia, orthostatic hypotension, and deep venous thrombosis [5–7]. Current research suggests that preadmission functional status, severity of illness, and comorbidities are predictors of survival in the ICU [8]. Despite the evidence that early mobilization and physical therapy are beneficial to critical care patients, minimal research has been conducted examining the feasibility of early mobility protocols for intensive care patients with nursing staff implementing these protocols [9]. With the increasing evidence supporting the use of early mobility in critically ill patients, it is important to establish a protocol that is beneficial for patients and can be easily implemented by nursing staff. 

While the mean length of stay is currently 3.86 days in an ICU environment, critical care patients who are at risk for immobility often require prolonged hospital stays [10]. These patients are often mechanically ventilated, confined to the bed, and sedated, which, in addition to their acute illness, contributes to the deconditioning of multiple organ systems [11, 12]. This deconditioning can occur in a few days of inactivity with some reports indicating that critically ill patients can lose up to 25% peripheral muscle weakness within 4 days when mechanically ventilated and 18% in body weight by the time of discharge [13–15]. Loss of muscle mass particularly skeletal muscle is higher in the first 2-3 weeks of immobilization during an intensive care unit stay [16, 17]. 

Muscle weakness and wasting were the most prominent complications reported by critical care patients who survive their intensive care stay, resulting in persistent functional disability in patients evaluated after discharge [6, 17, 18]. Weakness [4] and delirium [5] are associated with mechanical ventilation and ICU stays, leading to major functional status debility and lengthening rehabilitation. In fact, critically ill patients who are on strict bed rest have a decline of 1% to 1.5% per day and up to 50% of total muscle mass in 2 weeks [13].

Weakness and delirium are associated with mechanical ventilation and ICU stays, leading to major debility and longer rehabilitation [19]. Ventilator-associated pneumonia (VAP) occurs in 9 to 27 percent of ventilated patients with mortality rates between 33 and 55 percent in affected patients [20, 21]. Up to 60% of discharged critically ill patients may have long-term complications inhibiting them from complete functional recovery [22]. 

Early mobilization of patients in an intensive care unit, in addition to daily wakeups, and spontaneous breathing trials can enhance functional status, increase recovery, time and decrease hospital stay. In a prospective study of 347 critical care patients who received mechanical ventilation for 14 days or more, half of the patients had moderate functional impairment [1]. Participants also reported an increase of pain as well as problems with pain, sleep energy, mobility, and respiratory status in relation to their functional status [1].

Another study examining 90 critically ill patients determined that an early mobility protocol would enhance functional recovery and concluded that intensive care patients could benefit from early exercise. This randomized control trial exposed critically ill patients to a bedside cycle ergometer after 5 days for 20 minutes a day [15]. Patients in the treatment group tended to walk independently at hospital discharge compared to patients in the control group (73 versus 55) and were least likely to be referred to an outpatient rehabilitation center at discharge (17% versus 10%). In addition to the increased mobility at discharge, this study showed that an early mobility protocol could be initiated in an intensive care unit safely. Another study initiated a patient mobility protocol in a respiratory care unit within 48 hours of mechanical ventilation. These patients followed an activity protocol (sitting in bed, sitting in chair, and ambulation) that was initiated in an intensive care unit and ended with discharge to a step down unit or medical floor. Although this mobility protocol was recognized to be safe and had an improvement of outcomes (decreased hospital stay and intensive care days), the protocol itself was not diagrammed or standardized for nurses to follow [20].

Another study examined the impact of early exercise during sedation interruption. Patients were randomized to two groups following >72 hours of ventilation. Patients in the treatment group (*n* = 49) were given physical and occupational therapy during their daily wakeup. Patients in the control group (*n* = 55) were provided with the standard of care. Patients in the treatment group were more likely to return to independent functional status (59% versus 35%, *P* = 0.02), shorter incidence of delirium (28% versus 41%, *P* = 0.01), and more ventilator-free days (3 days versus 6 days, *P* = 0.02) [19].

The duration and frequency of activity that a critically ill patient experiences can have a significant impact on outcomes. To define this impact, one study used an actigraph to record the amount of mobility that a critically ill patient undergoes in an 8-hour period. Twenty chronically ill patients were studied during an 8-hour shift, and the frequency of their movements was recorded. Patients received only 64 minutes of movement in an 8-hour shift and were only turned 3 times compared to the recommended 4 times [7]. This suggests that ICU patients have infrequent activity and short duration of therapeutic activity and warrants the use of a mobility protocol to ensure that adequate movement is occurring [7]. Due to this variability of activity, a nurse-led mobility protocol would be ideal as the nurse could provide activity to the patient at any time, in turn maximizing patient readiness for activity.

Recently, a new approach to improve critical care patient outcomes has been proposed using evidence-based practice. This bundle includes an awakening and breathing coordination, delirium monitoring and management, and early mobility protocol that can be used in everyday clinical practice [23]. To ensure successful implementation, this bundle requires leadership, communication, and independence from trained staff to adapt these protocols to specific critical care environments. The nurse is key to successful implementation of these critical care bundles or protocols in an intensive care unit as the communication connection between patients and physicians. The use of effective bundles require daily consideration for every critical care patient in the critical care unit due to potential patient condition changes. Using evidence-based practice bundles such as the ones Balas et al. (2012) devised can assist critical care teams to mobilize their patients, leading to prevention or decrease in delirium and weakness [23].

**Figure 1:**
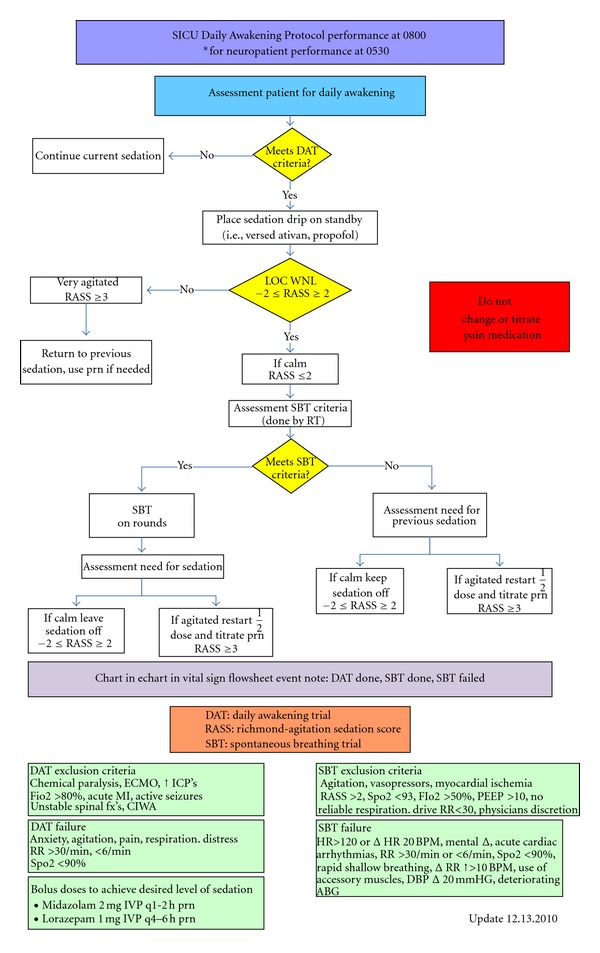
Daily awakening protocol.

Although current research has established the importance of daily awakenings, spontaneous breathing trials, and delirium/sedation management along with mobility protocols, very little research discusses the use of early mobility protocols to improve patient outcomes, and there are many remaining gaps in the literature. The protocols that are available in the literature do not provide enough details for replication. This is especially concerning since the importance of mobility has been established in the literature. Mobility protocols are so important that Critical Care Clinics (2007) devoted an entire issue addressing the barriers and facilitators of protocol intervention. In 2010, Critical Care Nurse provided a supplement addressing progressive mobility in the critically ill [24]. Despite this importance, current mobility research is limited with no published protocols of how these programs should be implemented in the critical care setting. In fact, while all patients in the surgical intensive care unit for this study were placed on a daily awakening protocol as well as a protocol for sedation, delirium, and analgesic management, a formal early mobility protocol had not been designed (Figure 1). Therefore, the purpose of this study was to pilot an early mobility protocol to test the safety and feasibility for mechanically ventilated patients in the surgical-trauma intensive care unit in conjunction with our current standards of assessment.

## 2. Methods

This pilot study consisted of two phases, protocol development and piloting of the protocol for use with ventilator-dependent surgical intensive care patients. Approval for this pilot study was received from the appropriate institutional review board. Research questions included (1) is an early mobility protocol safe and feasible for surgical-trauma intensive care patients? (2) is there a decrease in ventilator days or length of stay for patients using an early mobility protocol? and (3) what are the effects of an early mobility protocol on vital signs and perceived effort? Three patients consented to participate in this pilot study with outcomes examining protocol efficacy (adverse events, ease of use), patient perception of exertion after exercise, and length of stay. 

Data collection from patients was continued from the time of patient consent until the patient was discharged from the SICU. Patient data included demographics, admission and discharge date, diagnosis, comorbidities, amount of ventilator days, vital signs, and perceived exertion score. The activity level and determination for protocol advancement were determined by the physical therapist and attending physician on the research protocol. Data analysis consisted of calculating the mean scores and standard deviation for perceived exertion scale, vital signs, length of stay, and ventilator-free days. Efficacy was determined by calculating the number of adverse events for patients on the protocol.

### 2.1. Sample

For this pilot study, surgical-trauma intensive care patients were approached for consent after their cognitive status was deemed appropriate by the attending physician. Additional inclusion and exclusion criteria are presented in Table 1. 

### 2.2. Setting

The study setting was the SICU at a large academic teaching hospital in the Southeastern United States. This 16-bed unit is dedicated to the care of critically ill surgical and level-one trauma patients. The SICU has 24-hour nursing, respiratory therapy, and physician/nurse practitioner coverage. Physical therapists round on every patient with a consult in the SICU and participate in activities as determined by the therapist. 

### 2.3. Procedures

This pilot study consisted of two phases, development of the mobility decision-making tree and piloting of the protocol to determine efficacy of use in mechanically ventilated SICU patients. Starting with Stiller's safety mobilization guidelines (Table 2) for ICU clinicians, a multidisciplinary team was formed consisting of critical care nurses, nurse managers, a clinical nurse specialist, attending physician, physical therapist, and respiratory therapist to develop a mobilization protocol [25]. The protocol was developed based on the literature reviewed and feedback regarding feasibility and efficacy by members of the research team. The multidisciplinary team met 4 times to discuss the protocol and make revisions before subjecting the protocol to pilot study. The protocol was piloted to determine its feasibility, ease of use, and applicability for surgical intensive care patients. 

The protocol was comprised of 6 activity events. The activities are numbered from 1–6 (number 1 bed in chair position, number 2 sit on side of bed, number 3 stand at side of bed with 2 staff members, number 4 stand at side of bed, weight shift, minisquats, single-leg march, lateral steps along length of bed, number 5 walk 5 feet to chair with assistance, number 6 and walk 50–100 feet with staff assistance). After education about the mobility protocol to the SICU staff, the protocol was then pilot tested on three patients in the Surgical-Trauma ICU. 

The investigators spoke with the SICU charge nurse every day to determine any eligible SICU patients. All potential study patients were cleared and approved by their primary team attending. Once clearance and patient consent were obtained mobility progression was started by the bedside nurse and a member of the research team twice a day (10 AM and 2 PM) until discharge from the SICU. All patients began with level one on the mobility protocol.

The role of the nurse during this time was to monitor vital signs (blood pressure, heart rate, and oxygen saturation) and to insure that all lines and tubes were secure. Data was collected by a member of the research team at baseline, at completion of the activity, and 15 minutes following completion of the activity. Data was secured on a Microsoft Excel sheet and locked in the investigator's office when not in use. Since this is a pilot for efficacy and only three patients were consented, it was not possible to achieve statistical significance. Therefore, means and standard deviations were calculated for each outcome variable as appropriate.

### 2.4. Measures

Patient demographics, admitting diagnosis, and co-morbidities were collected for the pilot study participants. The following measures were collected as outcome variables to determine the effectiveness of the practice-derived decision tree for mobility.

#### 2.4.1. Perceived Exertion Scale

**Figure 2:**
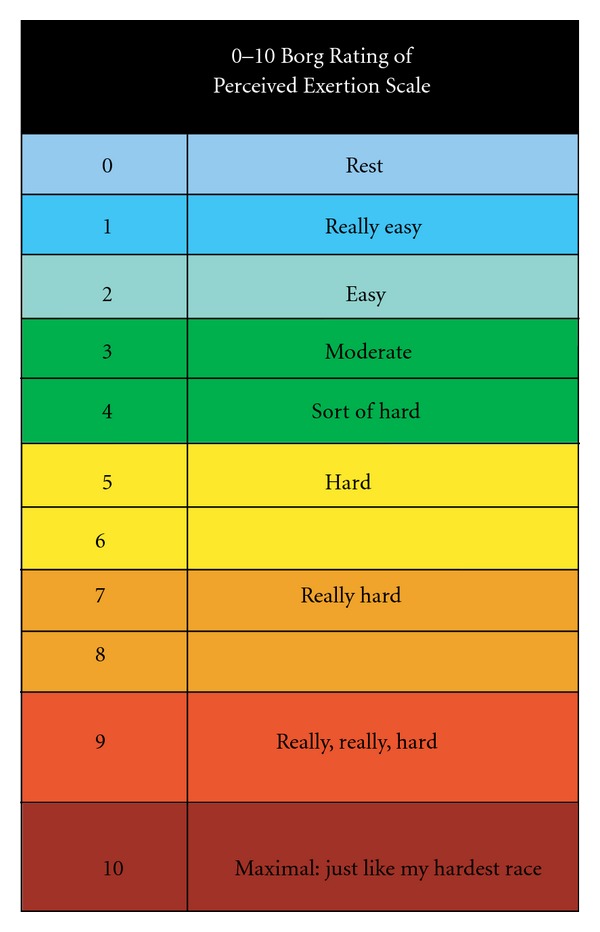
Borg exertion scale.

The Borg Rate of Perceived Exertion (Figure 2) was used to evaluate the patient's perceived fatigue level before and after the intervention [26, 27]. The Borg Rate of Perceived Exertion is designed to describe perceptions of physical exertion during a wide range of exercise modes. The scale consists of numbered categories, 0–10, and verbal cues, from “very, very light” to “very, very hard.” Reliability of this tool has been strong in patient populations and ranges from .66 to .78 [28]. 

#### 2.4.2. Vital Signs

Vital signs included the patient's blood pressure, heart rate, respiratory rate, and pulse oxygenation at baseline, five minutes after completion of the mobility intervention and 15 minutes after completion of the activity.

#### 2.4.3. Length of Stay

Length of stay was measured by the number of days the individual remained in the surgical intensive care unit.

#### 2.4.4. Ventilator-Free Days

Ventilator-free days were measured as the number of calendar days that the patient was not on the mechanical ventilator. Patients were considered “ventilator-free” if they were permanently placed on a tracheotomy collar.

## 3. Results

The protocol was modified two times as a result of the feedback from the interdisciplinary team before the protocol was pilot tested. The interdisciplinary team suggested including Synchronized Intermittent Mandatory Ventilation (SIMV) mode in addition to Pressure Support as criteria for advancing to Activity Level 5. For Activity Level 6, the minimum oxygenation saturation requirement was reduced from 94% to 93% as the team felt that 93% was a more acceptable saturation for the typical surgical-trauma intensive care patient.

**Figure 3:**
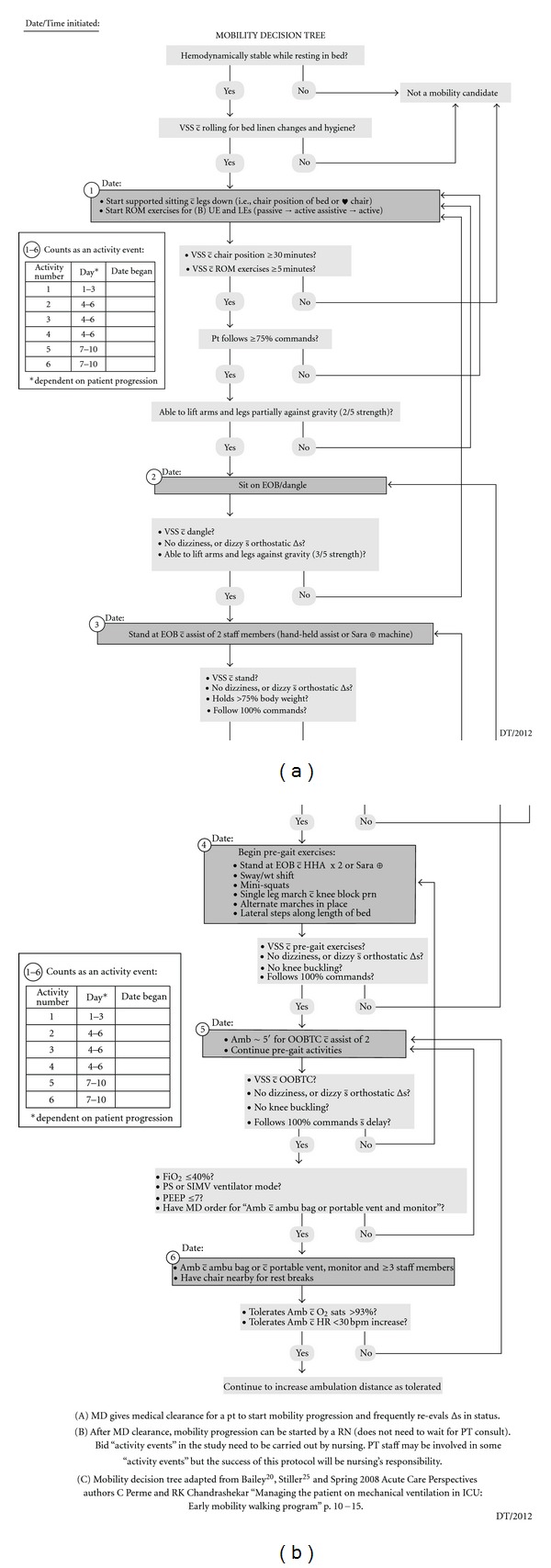
Final mobility protocol.

Following implementation with the pilot participants, the decision was made to relax the parameter for transitioning from Activity Level 1 to Activity Level 2 from 3/5 muscle strength in both arms and legs to 2/5 muscle strength in all extremities. The decision to alter the protocol was made based on observation of the patients and clinical judgment of the research team. The research team determined that participants would be ready to progress to the second activity at an earlier pace than the original protocol anticipated. The final mobility protocol is presented as Figure 3. 

### 3.1. Early Mobility Pilot Study

Participants in the pilot study ranged in age from 55 to 70 with two females and one male. Diagnoses were perforated diverticulitis, sepsis, and trauma post motor collision. APACHE scores were 15, 5, and 7, respectively. Comorbidities of the sample included hypertension, coronary artery disease, diabetes, high cholesterol, peripheral vascular disease, cancer, and anemia. 

#### 3.1.1. Efficacy

In terms of efficacy, two of the three patients completed the mobility protocol to Activity number 6 before discharge from the SICU and were successful ambulating using a tracheotomy collar or the portable ventilator. No adverse events (extubation, and line removal) were reported for these two patients. The remaining patient only completed Activity number 1 during her hospitalization in the SICU. 

#### 3.1.2. Perceived Exertion Scale

The average perceived exertion scores for the activity levels are presented in Table 3.

#### 3.1.3. Vital Signs

Vital signs (heart rate, blood pressure, respiratory rate, and oxygen saturation) remained stable immediately following and 15 minutes after activity completion for all three participants. 

#### 3.1.4. Length of Stay

The mean length of stay in the ICU was 13 days with participant length of stay being 8, 26, and 7 days, respectively. The average length of stay for patients in the SICU is 7 days.

#### 3.1.5. Ventilator-Free Days

Patients in this pilot study had an average of 7 ventilator-free days. Participant number 1 had 12 ventilator-free days, Participant number 2 did not have any ventilator-free days, and Participant number 3 had 8 ventilator-free days. The average ventilator-free days for patients in the SICU are 13 days.

## 4. Discussion

The purpose of this study was to develop and evaluate a mobility protocol. The efficacy of this pilot study was established and revisions were made to the protocol to allow ease of use. The nurses and physical therapists agreed that the protocol was easy to use due to the flowchart style and the decision tree matched the outcomes assessed by physical therapy. Two of the three pilot participants completed all activity levels with the third only achieving the first level. This patient was also responsible for the deviation in the length of stay variable (26 days) and did not have any ventilator-free days. The severity of this patient's illness contributed to the results of the study by increasing the average of the length of stay and ventilator-free day variables. 

The remaining participants were able to accomplish the protocol and be discharged from the intensive care unit. Length of stay values were less than the average SICU patient. Additionally, the patients' vital signs remained stable and no extubation or line removal events occurred. The implementation of this mobility protocol was successful within the constraints of this pilot study. Early mobilization is a priority of many intensive care nurses but there is often difficulty in setting up an established protocol for patient care. The results of this study suggest that nurses can incorporate a mobility protocol into the clinical setting, and thus achieve the bundle recommendations addressing awakening, delirium, ventilator management, and immobility [23]. 

## 5. Limitations

This was a pilot study to establish the efficacy and feasibility of a protocol for patient mobility. As such, the small sample size limits the interpretation and generalizability of these results. Future work will focus on determining if positive patient outcomes can occur for patients enrolled in this protocol. Since efficacy of the protocol has been established, the next step will be to conduct a randomized clinical trial to determine if the protocol improves clinical outcomes for Surgical-Trauma ICU patients.

## 6. Conclusions

Critically ill patients have limited activity due to their diagnosis, equipment, and condition. As a result of limited activity, they can suffer from deconditioning, muscle weakness, and infections. Physical deconditioning from their intensive care environment can occur after a few days in the unit. Presently, there is no gold standard for patient mobilization in a critical care environment. Therefore, future work will consist of further testing of the mobility protocol to determine if early mobilization in the ICU improves patient outcomes. Using the results of this pilot study as a guide, our research team is implementing a randomized study to compare outcomes of SICU patients using the early mobility protocol versus the standard of care. Results from this study will help determine if a patient mobility protocol is effective in the intensive care setting.

Beyond the significant physiological changes associated with constrained activity, our pilot study illustrates the multidisciplinary role needed to develop a useful nursing protocol. Future work should focus on the outcomes of implementing a mobility protocol in the intensive care environment, as patients may avoid the detrimental sequelae of an intensive care environment.

## Figures and Tables

**Table 1:** Inclusion/exclusion criteria for pilot study.

Inclusion criteria	Exclusion criteria
The patient must understand spoken English	The patient is unable to understand spoken English
The patient must reach a level of mentation that permits interaction with staff	The patient has pelvic, long bone fractures or is in skeletal traction
The patient must have been intubated for a minimum of 72 hours	The patient has been intubated for <72 hours (patients are at greater risk for physical debilitation after 72 hours)
The patient is physiologically stable (no pressors, vital signs wnl)	The patient is on full spine precautions
The patient will have no invasive femoral arterial lines	The patient has a head injury such as acute traumatic brain injuries and/or increased intracranial pressure
The patient is being cared for in the surgical/trauma intensive care unit	The patient does not meet the respiratory criteria of Fio2 < 60%, rate < 12, PEEP < 10 and O_2_ sats >94
Patients on tracheotomy collar trial Fio2 less than 60% with at least 2 hours on the ventilator during a-24 hour period	The patient has orthostatic hypotension
	The patient has facial trauma or known difficult airway
	The patient has a BMI >40
	The patient has evidence of metastatic lung disease

**Table 2:** Stiller safety considerations for mobilizing critically ill patients (2007).

(1) Safer to increase the intensity of activity slowly and progressively as each treatment is tolerated	
(2) General physiological principle and clinical acumen guide clinical practice	
(3) Activity should be selected based on assessment of patient's underlying cardiovascular and respiratory reserve	
(4) Activity should be determined from the patient's response to previous mobilization treatments	
(5) Appropriate activity duration and frequency are extremely variable for critically ill patients	
(6) Duration and frequency depend on patient's underlying condition	
(7) Mobilization should be functional as possible	
(8) If possible, a short warm-up period should be accomplished	
(9) Patient safety should be considered during all phases of a mobilization activities	

**Table 3:** Average perceived exertion scores.

Activity level	Average perceived exertion scores (SD)	
	Immediately postactivity	15-minutes postactivity
1	5 (SD = 1)	3 (SD = 1)
2	5 (SD = 1)	4 (SD = 1)
3	6 (SD = 1)	5 (SD = 1)
4	6.6 (SD = 2)	4.5 (SD = 1)
5	4.8 (SD = 2.5)	2.8 (SD = 0.4)
6	4.5 (SD = 3.7)	2.5 (SD = 2.1)
